# Prevalence, genotype profile and risk factors for multiple human papillomavirus cervical infection in unimmunized female adolescents in Goiânia, Brazil: a community-based study

**DOI:** 10.1186/1471-2458-13-1041

**Published:** 2013-11-04

**Authors:** Rosane Ribeiro Figueiredo Alves, Marília Dalva Turchi, Lyana Elias Santos, Eleuse Machado de Britto Guimarães, Mônica Maria Danda Garcia, Mirian Socorro Cardoso Seixas, Luisa Lina Villa, Maria Cecília Costa, Marise Amaral Rebouças Moreira, Maria de Fátima da Costa Alves

**Affiliations:** 1 Department of Obstetrics and Gynecology, School of Medicine, Federal University of Goiás, Goiânia, Brazil; 2 Institute of Tropical Pathology and Public Health Federal University of Goias, Postgraduate Studies Program, Goiânia, Brazil; 3 Division of Adolescent Medicine, Department of Pediatrics, School of Medicine, Federal University of Goiás, Goiânia, Brazil; 4 Department of Radiology and Basic Oncology, School of Medicine, University of São Paulo and Center for Translational Investigation in Oncology, ICESP, São Paulo, Brazil; 5 Department of Pathology, Radiology and Diagnostic Imaging. School of Medicine, Federal University of Goiás, Goiânia, Brazil; 6 Rua 235 S/N Setor Universitário, Goiânia, Goiás, Brazil

**Keywords:** Epidemiology, Multiple HPV infection, PCR-PGMY09/11/line blot hybridization/RFLP, Adolescents, female, Cytological abnormalities

## Abstract

**Background:**

The epidemiology of infection with multiple human papillomavirus (HPV) types in female adolescents is poorly understood. The purpose of this study was to explore the epidemiology of infection with multiple HPV types in adolescents and its association with demographic, behavioral and biological variables, as well as with cytological abnormalities.

**Methods:**

This community-based study included 432 sexually active females between 15 and 19 years of age. Genotyping for 30 HPV types was performed using a reverse blot strip assay/restriction fragment length polymorphism. Unconditional multivariate logistic regression was performed to identify factors significantly associated with HPV infection. The association between HPV infection and cytological abnormalities was calculated using a prevalence ratio.

**Results:**

The most common HPV types detected were 16, 51, 31, 52 and 18. Of the 121 HPV-positive women, 54 (44.6%) were infected with multiple HPV types. Having more than one lifetime sexual partner was associated with infection with any HPV infection, single HPV infection, and infection with multiple HPV types. The presence of cytological abnormalities was associated with infection with multiple HPV types.

**Conclusions:**

Co-infecting HPV genotypes occur in a high proportion of sexually active adolescents. Socio-demographic or sexual behavior factors associated with single HPV infection were similar to those associated with multiple HPV types. The higher risk of cytological abnormalities conferred by infection with multiple HPV types suggests a potential role of co-infection in the natural history of HPV infection.

## Background

Human papillomavirus (HPV) cervical infection is one of the world’s most common sexually transmitted infections, and is also a well-established cause of cervical cancer [1,2]. The highest prevalence of single or co-infection with multiple HPV types has been found in sexually active adolescents, young women [3-7], and women with impaired immune responses [8].

Studies of the dynamics of multiple HPV infections suggest that the acquisition of different HPV types occurs more often than expected by chance, and shared risk factors may explain the higher frequency of co-infections [9,10]. According to previous studies, the risk factors for cervical infection with multiple HPV types among HPV-infected women share several similarities with risk factors for HPV infection in general [6,7]. In addition, previous studies suggest that *Chlamydia trachomatis* infection can provide target host cells for the acquisition of HPV and may enable persistent HPV infection [6,11].

HPV infections and cytological abnormalities are common in adolescents, appearing shortly after the onset of sexual activity. More than 90% of HPV infections and cytological abnormalities regress within 3 years [12,13]. Co-infection with multiple HPV types has been associated with longer infection duration and a higher risk of cytological abnormalities and cervical neoplasia [3,14-16]. However, a consensus concerning these associations has not been reached [2,5,17].

Data on HPV infection and cytological abnormalities in adolescents derived from non-healthcare settings are scarce, especially in South America [18,19], and particularly in the mid-western region of Brazil [20]. The recent availability of a vaccine against HPV infection is a promising primary prevention measure to reduce the burden of cervical cancer. Therefore, a description of the genotype distribution of multiple HPV infections and the prevalence and degree of cytological abnormalities in sexually active adolescents is necessary for characterizing the target population for this vaccine as well as reinforcing the recommendation for cervical cancer screening in this age group.

The objectives of this study were to estimate the prevalence, describe the genotype profile and identify the risk factors for multiple HPV infections among healthy adolescents in the central part of Brazil, prior to the implementation of a large-scale HPV immunization program. Additionally, the study aimed to investigate the potential association between multiple HPV infections and cytological abnormalities among adolescents from a non-healthcare setting.

## Methods

### Study design and setting

This was a cross-sectional, community-based study. The design and population characteristics of this study have been described previously [21]. Briefly, this study was conducted between 2002 and 2003 in the Northwest sector of Goiânia, a city of 1,093,007 inhabitants in the central part of Brazil. The overall population of the Northwest sector was approximately 160,030 inhabitants. During the study period, a total of 4,091 females aged 15–19 years were registered at the local Public Family Health Program.

### Study population and sampling

Households with potential participants were randomly selected from census information provided by the local health department using a systematic sampling scheme. All female adolescents in each selected household were invited by letter to attend the health center nearest to their residence. Among the 914 adolescents who accepted the invitation, 472 (51.6%) were sexually active. The adolescents were eligible if they were between 15 and 19 years of age, were not currently pregnant or postpartum, had not used oral or vaginal antimicrobial drugs in the previous 15 days and had not engaged in sexual intercourse in the previous 48 hours. In total, 432 adolescents fulfilled the requirements, answered an interviewer-administered questionnaire and had a gynecological examination.

All participants provided written informed consent to participate in the study. The study was reviewed and approved by the Ethics Committee on Human and Animal Medical Research of the University Hospital, Federal University of Goiás.

### Data collection

The data were obtained through an interviewer-administered questionnaire addressing sociodemographic characteristics (age, education, marital status), tobacco smoking habits, as well as the subject’s sexual, gynecological and reproductive history, including age at menarche, age at first intercourse, interval in years between the age of first intercourse and the age of menarche, lifetime number of sexual partners, number of new sexual partners in the previous 3 months, frequency of condom use, history of full-term pregnancies and age of first pregnancy.

The cervical samples collected during the gynecological examinations were used for Papanicolaou tests, HPV and *C. trachomatis* DNA detection. Ectocervical and endocervical cells were collected with a cytobrush, which was then immersed in 10 mM Tris (pH 7.4 plus 1 mM EDTA), refrigerated and transported to the laboratory of Tropical Pathology and Public Health, Federal University of Goiás, and stored at −80°C until analysis to detect HPV and *C. trachomatis*.

*C. trachomatis* DNA detection was performed by PCR with primer pairs CP24 and CP27, which amplify a 207 nucleotide sequence of the cryptic plasmid*.* Positive and negative controls were included in each assay. An internal control was conducted in each amplification reaction to detect inhibitors. The assay was performed at the Department of Immunology, Tropical Pathology and Public Health Institute/Federal University of Goiás.

Papanicolaou smears were transported to and interpreted in the Pathology and Anatomy University Laboratory, in accordance with the 2001 Bethesda System [22]. Cytology assessments were conducted blind to the results of the exposure status.

### Detection of specific HPV types by polymerase chain reaction (PCR)/reverse blot strip assay/restriction fragment length polymorphism (RFLP)

DNA was extracted using phenol chloroform and amplified using PGMY09/11 HPV specific primers that amplify a 450-bp fragment of the L1 open reading frame of genital HPV [23]. DNA typing with a reverse line blot assay (Roche Molecular Systems) permitted the identification of 27 individual genital HPV types (6, 11, 16, 18, 26, 31, 33, 35, 39, 40, 42, 45, 51, 52, 53, 54, 55, 56, 57, 58, 59, 66, 68, 73, 82, 83 and 84). The assay was performed following the manufacturer’s guidelines at the Virology Unit of the São Paulo Branch of the Ludwig Institute for Cancer Research. RFLP analysis of the same 450-bp PCR fragment was also undertaken for some specimens [24]. DNA isolated from HPV-16 positive SiHa cells was used as a positive control, while negative controls were included to monitor potential PCR contamination. To confirm the integrity of the DNA material extracted from the specimens, assays included an additional set of primers (GH20 and PC04) to amplify a 268-bp region of the β-globin gene.

### Sample size and statistical analysis

A sample size of 430 participants was calculated to provide enough power to estimate a 10% prevalence of multiple HPV infections, with a precision of 3.0% and confidence interval of 95%, with a design effect of 1.2.

The prevalence of single HPV infection and multiple HPV infections (detection of more than one HPV type per sample) and their respective 95% confidence intervals (CIs) were calculated. Socio-demographic and sexual behaviors variables that were normally distributed were presented as mean and standard deviation (sd) and were categorized based on their mean values. Variables that were asymmetrically distributed were presented as median and interquartile range (IQR, Q1–Q3). Dichotomization of asymmetric variables was based on their median value. Years of schooling were dichotomized at 8 years, corresponding to the elementary Brazilian school period.

Potential risk factors for HPV infection were assessed using univariate and multivariate logistic regression analyses. Three different outcomes were analyzed: any HPV infection, single HPV infection, or infection with multiple HPV types. The three outcomes were each compared with non-infected participants, by calculating unadjusted and adjusted odds ratios (ORs) with respective 95% CIs. Variables with p < 0.20 in univariate analysis were included in multivariate models. Participants’ age was included in all models as a continuous variable, because age could be a confounding factor for HPV infection.

The association between HPV infection and cytological abnormalities was calculated using prevalence ratios and respective 95% CIs. All analyses were conducted with SPSS, version 16 for Windows. Differences were considered to be statistically significant when p < 0.05.

## Results

### Baseline characteristics

In total, 432 adolescents fulfilled the inclusion criteria and were interviewed. The participants’ mean age was 17.2 years, with a standard deviation of 1.3 years; approximately 88.0% were from low income families, 67.4% were unmarried, 57.3% reported 8 years of schooling or less, and 11.6% reported tobacco use. First sexual intercourse occurred at or before 15 years of age for 61.1% of the adolescents in this study. Almost 80.0% of participants reported inconsistent use of condoms by their partners and 46.0% reported more than one lifetime sexual partner. Furthermore, 41.7% of participants reported a previous pregnancy, with 19.0% conceiving before they were 15 years old (Table 1).

**Table 1:** Characteristics of participants

**Characteristics**	**Values**
Age (years)	
Range	15 to 19
Mean (sd)	17.2 (1.3)
Education (years) ^a^	
≤ 8 years (%)	247 (57.3)
> 8 years (%)	184 (42.7)
Marital status	
Unmarried (%)	291 (64.4)
Married (%)	141(35.6)
Family income (minimum wages)^b^	
≤ 4 (%)	359 (88.0)
> 4 (%)	49 (12.0)
Smoking	
Yes (%)	50 (11.6)
Age at menarche (years)	
Range	9 to 17
Mean (sd)	12.5 (1.3)
Age at first intercourse (years)	
Range	10 to 19
Mean (sd)	15.1 (1.5)
Number of lifetime sexual partners	
Range	1 to 20
Median (IQR Q_1_-Q_3)_	1 (1–3)
New sexual partner in past 3 months	
Yes (%)	63 (14.6)
Number of sexual partner in past 3 months	
Range	1 to 5
Median (IQR Q_1_-Q_3_)	1 (1–1)
Condom use	
Never/occasional (%)	345 (79.9%)
Always (%)	87 (20.1)
History of full-term pregnancies	
Yes (%)	180 (41.7)
Age at first full-term pregnancy (years) ^c^	
Range	12 to 19
Mean (sd)	15.6 (1.4)
*Chlamydia trachomatis* infection (%)	60 (13.9)
Cytological abnormalities	
Overall (%)	67 (15.4)
ASC-US (%)	46 (10.6)
LGSIL (%)	20 (4.6)
ASC-H (%)	1 (0.2)

sd: Standard deviation; IQR, Q1-Q3 interquartile range; ^a^ one adolescent without information; ^b^ 24 adolescents without information; MW = 334 US$; ^c^ four adolescents without information; ASCUS: atypical squamous cells of undetermined significance; ASC-H, atypical squamous cells cannot exclude high-grade intraepithelial lesion; LGSIL: low-grade squamous intraepithelial lesion.

The prevalence of *C. trachomatis* infection was 13.9% (95% CI: 10.8–17.6). The prevalence of cytological abnormalities was 15.4% (95% CI: 12.3–19.4), with atypical squamous cells of undetermined significance occurring in 10.6% (95% CI: 8.0–14.0), low-grade squamous intraepithelial lesion occurring in 4.6% (95% CI: 2.9–7.2) and atypical squamous cells cannot exclude high-grade intraepithelial lesion occurring in 0.2% (95% CI: 0.0–1.5) of adolescents. No participant had high-grade squamous intraepithelial lesion or “atypical” glandular cells (Table 1).

### Prevalence and classification of HPV infection

HPV DNA was detected in cervical specimens of 121 of the 432 adolescents, resulting in an overall prevalence of 28.0% (95% CI: 23.9–32.5; Table 2). The number of HPV types detected per specimen ranged from 0 to 5. Among the 121 HPV-infected participants, a single type of HPV infection was detected in 67 individuals, whereas multiple HPV types were detected in 54 adolescents (54/432; 12.5%), representing 44.6% of the positive samples (Table 2).

**Table 2:** HPV prevalence in participants from central Brazil

	**HPV positive participants**		**Multiple HPV positive participants**	
	**n**	**% (95% CI)**	**n**	**% (95% CI)**
HPV	121	28.0 (23.9-32.5)	54	100
One HPV type	67	15.5 (12.3-19.4)	-	-
Multiple HPV types	54	12.5 (9.6-16.1)	-	-
HR-HPV	107	24.8 (20.8-29.2)	53	98.1 (90.1-100.0)
LR-HPV	32	7.4 (5.2-10.4	19	5.8 (3.6-9.0)
HPV 16	29	6.7 (4.6-9.6)	21	38.9 (25.9-53.1)
HPV 51	22	5.1 (3.3-7.7)	18	33.3 (21.1-47.5)
HPV 31	20	4.6 (2.9-7.2)	12	22.0 (12.0-35.6)
HPV 52	18	4.2 (2.6-6.6)	10	18.5 (9.3-31.4)
HPV 18	15	3.5 (2.0-5.8)	12	22.2 (12.0-35.6)
HPV 53	11	2.5 (1.3-4.6)	11	20.4 (10.6-36.5)
HPV 58	10	2.3 (1.2-4.4)	8	14.8 (6.6-27.1)
HPV 39	9	2.1 (1.0-4,1)	8	14.8 (6.6-27.1)
HPV 6	7	1.6 (0.7-3.5)	6	11.1 (4.2-22.6)
HPV 45	6	1.4 (0.6-3.2)	1	1.9 (0.0-9.9)
HPV 66	6	1.4 (0.6-3.2)	3	5.6 (1.2-15.4)
HPV 73	6	1.4 (0.6-3.2)	2	3.7 (0.5-12.7)
HPV 42	5	1.2 (0.4-2.8)	3	5.6 (1.2-15.4)
HPV 54	5	1.2 (0.4-2.8)	2	3.7 (0.5-12.7)
HPV 26	4	0.9 (0.3-2.5)	2	3.7 (0.5-12.7)
HPV 33	4	0.9 (0.3-2.5)	3	5.6 (1.2-15.4)
HPV 56	4	0.9 (0.3-2.5)	2	3.7 (0.5-12.7)
HPV 68	4	0.9 (0.3-2.5)	3	5.6 (1.2-15.4)
HPV 40	3	0.7 (0.2-2.2)	1	1.9 (0.0-9.9)
HPV 84	3	0.7 (0.2-2.2)	3	5.6 (1.2-15.4)
HPV 35	2	0.5 (0.1-1.8)	2	3.7 (0.5-12.7)
HPV 70	2	0.5 (0.1-1.8)	0	-
HPV 55	2	0.5 (0.1-1.8)	1	1.9 (0.0-9.9)
HPV 61	2	0.5 (0.1-1.8)	0	-
HPV 62	2	0.5 (0.1-1.8)	2	3.7 (0.5-12.7)
HPV 59	1	0.2 (0.0-1.5)	1	1.9 (0.0-9.9)
HPV 82	1	0.2 (0.0-1.5)	1	1.9 (0.0-9.9)
HPV 11	1	0.2 (0.0-1.5)	0	-
HPV 89	1	0.2 (0.0-1.5)	1	1.9 (0.0-9.9)
HPV 44	1	0.2 (0.0-1.5)	0	-

HPV: human papillomavirus; CI: confidence interval; n: number of participants; HR-HPV: high-risk HPV; LR-HPV: low-risk HPV.

**Figure 1:**
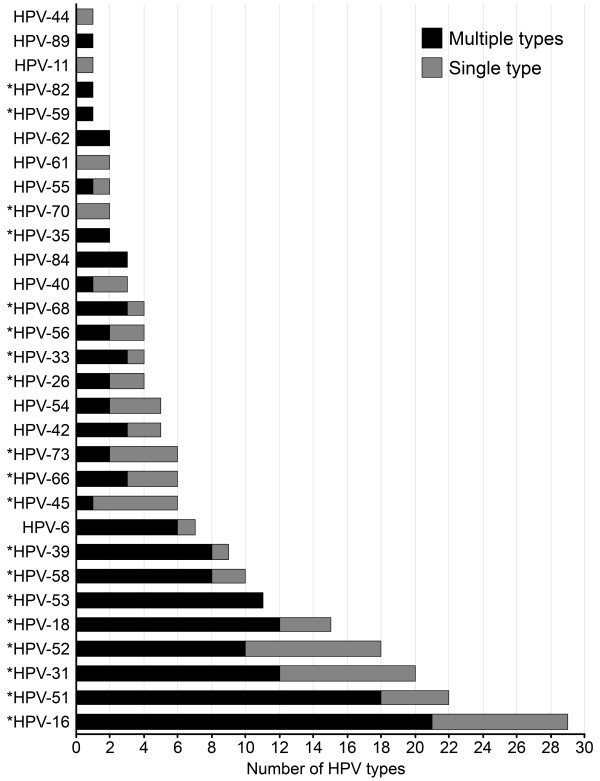
Absolute frequency of the 30 HPV types detected as single and multiple HPV infections (HPV: human papillomavirus; *oncogenic types; dark bars represent multiple HPV types; dotted bars represent a single HPV type).

Thirty different HPV types were detected; nineteen types were high-risk (HR)-HPV (16, 18, 26, 31, 33, 35, 39, 45, 51, 52, 53, 54, 56, 58, 66, 68, 70, 73 and 82), and 11 were low-risk (LR)-HPV types (6, 11, 40, 42, 44, 54, 55, 61, 62, 84, 89), according to the International Committee on the Taxonomy of Viruses [25]. Figure 1 shows the absolute frequency of the 30 different HPV types detected. These types were stratified by multiple (black bars) and by single (gray bars) infection (Figure 1). The prevalence of HR-HPV was 24.8% (CI: 20.8–29.2), which was higher than the LR-HPV type prevalence (Table 2).

The most prevalent HPV types were 16, 51, 31, 52 and 18, and the most common HPV types detected in multiple infections included 16, 51, 18, 31 and 52. The most common HPV type detected in single or multiple infections was HPV-16; HPV-6 was the most common LR-HPV detected (Figure 1). The prevalence of either HR-HPV type that is present in bivalent vaccines (HPV types 16 and 18) was 10.2%, while 12% of adolescents were infected with one of the types included in the quadrivalent vaccine (HPV types 6, 11, 16 and 18). After using both HPV DNA detection assays, only one specimen had an unclassified HPV type.

### Risk factors for HPV infection

Three different outcomes were analyzed: any HPV infection, infection with a single HPV type, and infection with multiple HPV types. The three outcomes were each compared to participants with no infections. The associations between baseline variables and HPV infection, as well as the results of the multivariate analyses for all variables retained in the final regression models, are presented in Tables 3, 4 and 5.

**Table 3:** Factors associated with HPV infection

**Variables**	**HPV infection**	**Total**	**(%)**	**OR (IC 95%)**	***p***	**Adjusted OR IC95%**	***p***
Age (years)							
≤ 17	69	237	29.1	1.1 (0.7 – 1.7)	0.57	-	-
> 17	52	195	26.7				
Education (years)^a^							
≤ 8	79	247	32.0	1.6 (1.0-2.5)	0.02	1.2 (0.6-2.2)	0.54
> 8	41	184	22.3				
Marital status							
Unmarried	93	291	32.0	1.9 (1.2-3.0)	<0.01	1.1 (0.5-2.1)	0.76
Married	28	141	19.9				
Smoking							
Yes	22	50	44,0	2.2 (1.2-4.1)	<0.01	1.7 (0.8-3.7)	0.15
No	99	382	25,9				
Age at first intercourse (years)							
10–15	80	264	30.3	1,3 (0,9-2,1)	0.18	0.8 (0.4-1.6)	0.59
16-19	41	168	24.4				
Age at menarche^b^ (years)							
13–17	56	207	27.1	0.9 (0.6-1.4)	0.70	-	-
9-12	64	223	28.7				
Condom use							
Never/occasional	98	345	28.4	1.1 (0.6-1.8)	0.71	-	-
Always	23	87	26.4				
Number of lifetime sexual partners							
> 1	88	199	44.2	4.8 (3.0-7.6)	<0,001	4.9 (2.5-9.5)	<0.01
1	33	233	14.2				
New sexual partner in past 3 months							
Yes	21	63	33.3	1.3 (0.7-2.4)	0.30	-	-
No	100	369	27.1				
Number of sexual partner in past 3 months							
> 1	12	21	57.1	3.7 (1.5-9.0)	<0.01	1.2 (0.3-4.2)	0.80
≤ 1	109	411	26.5				
Age at first full-term pregnancy (years) ^c^							
No	70	252	27.8	1	0.31	-	-
< 15	25	82	30.5	1.1 (0.6-1.9)	0.34	-	-
≥ 15	24	94	25.5	0.9 (0.5-1.5)			
*C. trachomatis* infection							
Yes	23	60	38.3	1.7 (1.0-3.1)	0.05	1.0 (0.5-2.2)	0.96
No	98	372	26.3				

HPV: *Human papillomavirus*; OR: odds ratio; CI: confidence interval; *C. trachomatis: Chlamydia trachomatis*. ^a^ one without information; ^b^ two without information; ^c^ Four without information. The variables “Education”, “Marital status”, “Smoking”, “Age at first intercourse”, “Number of lifetime of sexual partners” and the “Number of sexual partners in the past 3 months” were included in the multivariate model. The odds ratios have been adjusted for age as a continuous variable.

**Table 4:** Factors associated with single HPV infection

**Variables**	**Single HPV infection**	**Total**	**(%)**	**OR (IC 95%)**	***p***	**Adjusted OR IC95%**	***p***
Age (years)							
≤ 17	37	205	18.0	1.0 (0.6-1.8)	0.48	-	-
> 17	30	173	17.3				
Education (years)^a^							
≤ 8	41	209	19.6	1.4 (0.8-2.4)	0.11	1.1 (0.6-2.1)	0.70
> 8	25	168	14.8				
Marital status							
Unmarried	48	246	19.5	1.4 (0.8-2.6)	0.13	1.3 (0.6-2.8)	0.48
Married	19	132	14.4				
Smoking							
Yes	14	42	33.3	2.7 (1.3-5.4)	<0.001	1.4 (0.6-3.2)	0.37
No	53	336	15.8				
Age at first intercourse (years)							
10–15	44	228	19.3	1.3 (0.7-2.3)	0.19	0.8 (0.4-1.6)	0.53
16-19	23	150	15.3				
Age at menarche^a^ (years)							
13–17	29	180	16.1	0.8 (0.5-1.4)	0.21	-	-
9-12	38	197	19.2				
Condom use							
Never/occasional	52	299	17.4	0.9 (0.5-1.7)	0.42	-	-
Always	15	79	19.0				
Number of lifetime sexual partners							
> 1	18	218	8.2	2.9 (1.6-5.5)	<0.001	4.7 (2.4-9.1)	<0.001
1	49	160	30.6				
New sexual partner in past 3 months							
Yes	8	50	16.0	0.8 (0.4-1.9)	0.37	-	-
No	59	328	18.0				
Number of sexual partner in past 3 months							
> 1	12	61	7.4	1.2 (0.6-2.3)	0.32	-	-
≤ 1	55	317	17.3				
Age at first full-term pregnancy (years) ^b^							
No	34	216	15.7	1	0.12	1	0.81
< 15	19	89	21.3	1.4 (0.7-2.7)	0.36	0.8 (0.3-1.9)	0.63
≥ 15	12	69	17.4	1.1 (0.5-2.3)		0.6 (0.2-1.4)	
*C. trachomatis* infection							
Yes	11	48	22.9	1.4 (0.7-3.0)	0.27	-	-
No	56	330	16.9				

HPV: *Human papillomavirus*; OR: odds ratio; CI: confidence interval; *C. trachomatis: Chlamydia trachomatis*. ^a^ one without information; ^b^ Four without information. The variables “Education”, “Marital status”, “Smoking”, “Age at first intercourse”, “Number of lifetime of sexual partners” and the “Age at first full-term pregnancy” were included in the multivariate model. The odds ratios have been adjusted for age as a continuous variable.

**Table 5:** Factors associated with multiple HPV infection

**Variables**	**Multiple HPV infection**	**Total**	**(%)**	**OR (IC 95%)**	***p***	**Adjusted OR IC95%**	***p***
Age (years)							
≤ 17	32	200	16.0	1.3 (0.7-2.2)	0.28	-	-
> 17	22	165	13.3				
Education (years)							
≤ 8	38	206	18.4	2.0 (1.1-3.8)	0.02	1.8 (0.8-3.7)	0.11
> 8	16	159	10.0				
Marital status							
Unmarried	45	243	18.5	2.8 (1.3-6.0)	0.006	2.2 (0.8-5.6)	0.10
Married	9	122	7.4				
Smoking							
Yes	8	36	22.2	1.7 (0.7-4.1)	0.19	0.9 (0.3-2.3)	0.82
No	46	329	14.0				
Age at first intercourse (years)							
10–15	36	220	16.3	1.4 (0.7-2.5)	0.30	-	-
16-19	18	145	12.4				
Age at menarche^a^ (years)							
13–17	27	78	15.1	1.1 (0.6-1.9)	0.76	-	-
9-12	26	185	14.0				
Condom use							
Never/occasional	46	293	15.7	1.5 (0.7-3.3)	0..32	-	-
Always	8	72	11.1				
Number of lifetime sexual partners							
> 1	39	150	26.0	4.7 (2.4-8.9)	<0.001	3.7 (1.8-7.5)	<0.001
1	15	215	7.0				
New sexual partner in past 3 months							
Yes	13	55	23.6	2.0 (1.0-4.1)	0.04	0.6 (0.2-1.9)	0.42
No	41	310	13.2				
Number of sexual partner in past 3 months							
> 1	8	17	47.0	5.5 (2.1-15.9)	0.001	0.3 (0.1-1.1)	0.07
≤ 1	46	348	13.2				
Age at first full-term pregnancy (years) ^a^							
No	36	218	16.5	1	-	1	0.40
< 15	13	70	18.6	1.1 (0.6-2.3)	0.34	0.6 (0.3-1.6)	0.35
≥ 15	5	75	6.7	0.3 (0.1-0.9)	0.01	1.7 (0.5-5.7)	
*C. trachomatis* infection							
Yes	12	49		2.1 (1.0-4.4)	0.04	1.4 (0.6-3.2)	0.36
No	42	316					

HPV: *Human papillomavirus*; OR: odds ratio; CI: confidence interval; *C. trachomatis: Chlamydia trachomatis*. ^a^ two without information. The variables “Education”, “Marital status”, “Smoking”, “Number of lifetime of sexual partners”, “New sexual partner in past 3 months”, “Number of sexual partner in past 3 months”, “Age at first full-term pregnancy”, and “*C. trachomatis* infection” were included in the multivariate model. The odds ratios have been adjusted for age as a continuous variable.

Having a number of lifetime sexual partners greater than one was associated with a higher risk of any HPV infection (adjusted OR: 4.9 [95% CI: 2.5–9.5]) (Table 3), single HPV infection (adjusted OR: 4.7 [95% CI: 2.4–9.1]) (Table 4), and multiple HPV infection (adjusted OR: 3.7 [95% CI: 1.8–9.5]) (Table 5).

### Risk factors for cytological abnormalities

The prevalence ratio for cytological abnormalities between samples from adolescents infected with either one or multiple HPV types was calculated. Participants were categorized into either “negative for intraepithelial lesion or malignancy” or “epithelial cell abnormalities.” Participants with positive HPV results were then classified into non-overlapping categories, and the impact of multiple infections on cytological abnormalities (excluding HPV 16) was analyzed (Table 6). The prevalence ratio was 2.1 (95% CI: 1.1–4.0) for cytological abnormalities associated with a single HPV type, and was 6.4 (95% CI: 4.1–10.0) for multiple HPV types. The prevalence ratio when all adolescents infected with HPV-16 were excluded was 2.2 (95% CI: 1.3–4.3) for a single HPV type and 6.2 (95% CI: 3.7–7.1) for multiple HPV types.

**Table 6:** Association between cytological abnormalities and HPV infection (n = 432)

	**Cytological abnormalities**		**PR (95% CI)**	***p***
	**Positive**	**Total**		
HPV infection				
Negative	26	311	-	-
One type	12	67	2.1 (1.1-4.0)	0.02
Multiple types	29	54	6.4 (4.1-10.0)	<0.01
HPV infection without HPV-16				
Negative	26	311	-	-
One	11	59	2.2 (1.2-4.3)	0.03
Multiple types	17	33	6.2 (3.7-10.1)	<0.01

HPV: human papillomavirus; n: number; PR: prevalence ratios.

## Discussion

This community-based study investigated patterns of HPV infection in exfoliated cells from Brazilian adolescents according to HPV types. This study confirmed the high prevalence of HPV infection in sexually active adolescents. Moreover, it reaffirms the high rate of multiple HPV types, which agrees with previous studies involving sexually active adolescents and young women [4,6,14,26,27].

HR-HPV types were the most common HPV types detected (88.4%), and they were similar to the HPV types described in other studies [6,8,26,27]. HPV-16, in particular, was the most common type detected in cases of both single and multiple infections. The distribution of other HPV types differed from previous studies [4,26,27]. In the present study, HPV-18 was the fifth most prevalent type, behind types 16, 51, 31 and 52. More than one-third (36.4%) of HPV-positive specimens exhibited HPV-16/18 dual infection. Differences in the prevalence and distribution of the individual HPV types may be explained by the use of different methods used to detect HPV, variability in the clinical specimens used, the age of the participants and regional variation in the distribution of the types.

The HR-HPV types were detected in 80.6% of all single-type infections, and in 98% of all multiple-type infections. The high prevalence of multiple HPV types may be because high-risk types are more likely to cause persistent infections than low-risk types [4,12]. Furthermore, the high prevalence of multiple HPV types may be related to the increasing levels of estrogen exposure during pubertal development. Previous studies suggest that the increased levels of estrogen may modulate the immune response and facilitate cervical ectropion and squamous metaplasia, both of which add to the biological susceptibility of the uterine cervix [28,29].

The present study correlated HPV infection with the number of prior sexual partners, which produced results consistent with previously published reports [6,7,26], emphasizing once more the importance of sexual transmission. The highest prevalence of single or co-infection with multiple HPV types has been found in sexually active adolescents and young women [3-7]. However, it is uncertain whether the presence of multiple HPV types in adolescents is due to behavioral characteristics or whether adolescents with multiple HPV types possess certain intrinsic biological characteristics that increase susceptibility. The present study shows that the risk factors for multiple HPV infection were similar to the risk factors for any HPV infection and single HPV infection, which agrees with previous studies of sexually active adolescents and young women [6,7]. However, the cross- sectional design of this study will not be able to adequately answer that if the risk factors for multiple HPV infection were distinct from risk factors for any HPV infection. Indeed, HPV infections in sexually active teenagers come and go. Those classified as “single infection” during this study may well have had a multiple infections a few weeks prior, and vice versa.

The role of multiple HPV infections in cervical carcinogenesis is still controversial. Some studies have reported an association between multiple HPV infections and a higher risk of cytological abnormalities and cervical neoplasia [14-16], although these findings are not consistent [2,5,17], and these correlations are not associated with viral persistence, compared with infection due to one HPV type [9,17]. The present study shows a significant association between cytological abnormalities and detection of both single and multiple HPV types; however, this association was significantly stronger for infections with multiple HPV types, as reported in other studies [14,15].

This association could be explained by the elevated prevalence of HR-HPV types detected in samples with multiple infections in this study. HR-HPVs are often present as persistent infections, representing the main risk factor for the development of lesions [12]. However, there is some evidence that infection with multiple HPV types confers an increased risk for the development and/or progression of lesions, regardless of the persistence of HPV infections [15]. In contrast, the low-grade cytological abnormalities found in this study may reflect the viral load resulting from productive infections, many of which tend to regress over time, thereby resulting in less clearly identifiable lesions. Additionally, the excess risk of cytological abnormalities resulting from multiple infections remained even after excluding the adolescents who harbored HPV-16, a similar finding to the Ludwig-McGill cohort study [15]. The smaller sample size for multiple HPV infection, without those with HPV-16, may have limited the power of the study to detect large differences.

A potential limitation of this investigation was the reliance on cytological samples of cervical lesions without corresponding biopsy results. The cytological screening interpretation presents known problems with specimen collection, smear preparation and inter-observer reproducibility in all cytological categories [30,31]. The US multicenter clinical trial, “ASCUS-LSIL Triage Study” (ALTS), showed that inter-observer reproducibility of cytological interpretation of a large series of specimens was only moderate and the greatest source of disagreement involved interpretation of atypical squamous cells of undetermined significance (ASC-US), of which 37% were HPV positive [30]. In the present study, 61.0% of samples with cytological abnormalities and 47.8% of ASC-US samples were positive for HPV. This reflects careful cytological assessment in a reference laboratory following strict quality control guidelines. This strategy was adopted to avoid unnecessary biopsies for lesions that have a higher potential for spontaneous regression.

Another potential limitation of this study was the estimation of prevalence by cross-sectional detection of type-specific HPV infections, which may underestimate the cumulative cytological abnormalities resulting from exposure to different HPV types over time. Furthermore, this investigation relied on the accuracy of the memories of subjects for precise information about their sexual and reproductive history.

## Conclusions

The detection of any, single or multiple HPV types in cervical specimens of adolescents was associated with the number of lifetime sexual partners. These findings also show that in adolescents, infection with multiple HPV types is associated with the development of more cervical cytological abnormalities than infection with a single HPV type.

By providing data on the epidemiology of HPV infection with multiple HPV types in adolescents, these results may inform the planning and the interpretation of models evaluating the impact and cost effectiveness of vaccines. These estimates, obtained prior to the introduction of HPV vaccination, provide a baseline for monitoring and surveillance of the effectiveness of this primary prophylactic intervention in vaccinated and unvaccinated adolescents. The high rate of HPV detection in adolescents emphasizes the advantages of early vaccination, prior to the onset of sexual activity. Furthermore, the high prevalence of minor cytological abnormalities, which tend to regress with time, reinforces the recommendation not to include teenagers in cervical cytology screening programs. Although most cytological abnormalities in adolescents may regress over time, there is a need for longitudinal studies to assess the cumulative influence of infection with multiple HPV types in the progression of cervical lesions with increasing age.

## Abbreviations

AGC: Atypical glandular cells; ASC-US: Atypical squamous cells of undetermined significance; ASC-H: Atypical squamous cells, cannot exclude high-grade intraepithelial lesion; CI: Confidence interval; HGSIL: High-grade squamous intraepithelial lesion; HPV: Human papillomavirus; HR-HPV: High-risk HPV; LGSIL: Low-grade squamous intraepithelial lesion; LR-HPV: Low-risk HPV; OR: Odds ratio; PCR: Polymerase chain reaction; RFLP: Restriction fragment length polymorphism; sd: Standard deviation.

## Competing interests

LLV is a board member of Merck & Co.

## Authors’ contributions

EMBG and MFCA were responsible for the study design, coordination and data collection. LES, LLV and MCC carried out the molecular genetic studies. MARM performed cytological assessment. MMDG and MSCS participated in the data collection and the gynecological examinations. RRFA and MDT performed the statistical analyses and wrote the manuscript. All authors have read and approved this manuscript.

## Pre-publication history

The pre-publication history for this paper can be accessed here:

http://www.biomedcentral.com/1471-2458/13/1041/prepub
